# pyaging: a Python-based compendium of GPU-optimized aging clocks

**DOI:** 10.1093/bioinformatics/btae200

**Published:** 2024-04-11

**Authors:** Lucas Paulo de Lima Camillo

**Affiliations:** School of Clinical Medicine, University of Cambridge, Cambridge CB2 0SP, United Kingdom

## Abstract

**Motivation:**

Aging is intricately linked to diseases and mortality. It is reflected in molecular changes across various tissues which can be leveraged for the development of biomarkers of aging using machine learning models, known as aging clocks. Despite advancements in the field, a significant challenge remains: the lack of robust, Python-based software tools for integrating and comparing these diverse models. This gap highlights the need for comprehensive solutions that can handle the complexity and variety of data in aging research.

**Results:**

To address this gap, I introduce pyaging, a comprehensive open-source Python package designed to facilitate aging research. pyaging harmonizes dozens of aging clocks, covering a range of molecular data types such as DNA methylation, transcriptomics, histone mark ChIP-Seq, and ATAC-Seq. The package is not limited to traditional model types; it features a diverse array, from linear and principal component models to neural networks and automatic relevance determination models. Thanks to a PyTorch-based backend that enables GPU acceleration, pyaging is capable of rapid inference, even when dealing with large datasets and complex models. In addition, the package’s support for multi-species analysis extends its utility across various organisms, including humans, various mammals, and *Caenorhabditis elegans*.

**Availability and implementation:**

pyaging is accessible on GitHub, at https://github.com/rsinghlab/pyaging, and the distribution is available on PyPi, at https://pypi.org/project/pyaging/. The software is also archived on Zenodo, at https://zenodo.org/doi/10.5281/zenodo.10335011.

## Graphical Abstract

**Graphical Abstract:**
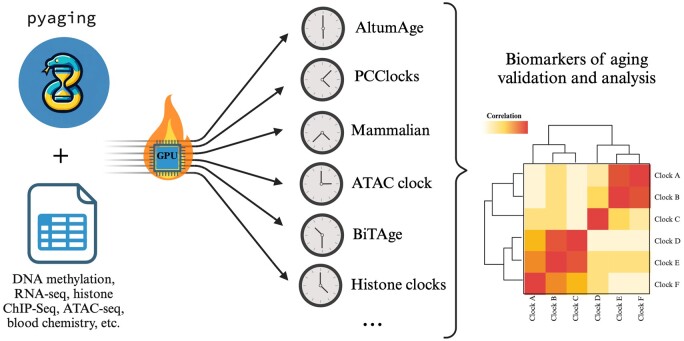

## 1 Introduction

As we entered the 21st century, longevity studies became the cornerstone of aging research in various model organisms. The span of these studies ranged from a few days in *Caenorhabditis elegans* to several weeks in Drosophila melanogaster, extending up to a few years in *Mus musculus*. This spectrum allowed for manageable daily mortality tracking in the burgeoning field of gerontology. Nonetheless, the feasibility of lifespan studies, both in terms of time and cost, remains a significant challenge. The transformative work by Horvath in 2013 marked a pivotal moment, introducing a reliable age predictor and catalyzing a new domain of research focused on the development and refinement of biomarkers of aging, healthspan, and lifespan (Horvath 2013).

Presently, the field boasts over a hundred aging clocks—machine learning models designed to predict various aspects of aging. DNA methylation, undoubtedly the most popular data type for constructing aging biomarkers, is complemented by other molecular signatures like transcriptomics, proteomics, blood chemistry, histone modification, and chromatin accessibility, each offering unique advantages. However, there exists a notable gap in software tools that consolidate these diverse aging clocks for comparative analysis. A few notable initiatives, such as the R packages methylclock (Pelegí-Sisó *et al.* 2021) and methylCYPHER (Thrush *et al.* 2022), represent steps toward addressing this need.

Yet, the development of aging biomarkers is not without its challenges, as underscored in a recent perspective (Moqri *et al.* 2023). Current software tools in this domain face several limitations: (i) while popular in biology, the prevalent use of R, an ad-hoc object-oriented programming system, often lacks the versatility needed for complex models like neural networks, which are more effectively implemented in languages such as Python; (ii) most existing age prediction packages are limited to a handful of clocks, far fewer than the actual breadth of available models; (iii) a focus predominantly on DNA methylation biomarkers narrows the scope for cross-comparison across different molecular layers; (iv) the lack of nonlinear techniques, such as neural network-based approaches like AltumAge (Galkin *et al.* 2021, de Lima Camillo *et al.* 2022); (v) the reliance on CPU processing results in slower inference, especially with larger datasets and more complex models; (vi) a species-specific focus, predominantly on *Homo sapiens*.

Addressing these challenges, I have developed pyaging, a Python-based package that acts as a comprehensive repository for various biomarkers of aging and aging clocks. pyaging offers: (i) a Python-centric approach, utilizing the versatile AnnData (Virshup *et al.* 2021) format of annotated data matrices in memory and on disk; (ii) an expanding repository, currently encompassing over 50 clocks with routine updates given new developments in the literature; (iii) clocks based on a diverse range of data types, encompassing DNA methylation (Hannum *et al.* 2013, Horvath 2013, Knight *et al.* 2016, Lin *et al.* 2016, Petkovich *et al.* 2017, Stubbs *et al.* 2017, Zhang *et al.* 2017, Horvath *et al.* 2018, Levine *et al.* 2018, Meer *et al.* 2018, Thompson *et al.* 2018, Lee *et al.* 2019, Lu *et al.* 2019, Zhang *et al.* 2019, Han *et al.* 2020, McEwen *et al.* 2020, Belsky *et al.* 2022, de Lima Camillo *et al.* 2022, Endicott *et al.* 2022, Higgins-Chen *et al.* 2022, Lu *et al.* 2022, Dec *et al.* 2023, Li *et al.* 2023, Lu *et al.* 2023, McGreevy *et al.* 2023, Ying *et al.* 2024), transcriptomics (Meyer and Schumacher 2021), histone mark ChIP-Seq (de Lima Camillo *et al.* 2023), and ATAC-Seq (Morandini *et al.* 2024); (iv) a variety of models, including linear, principal component (PC) linear models, neural networks, and automatic relevance determination (ARD) (MacKay 2003) models; (v) a PyTorch-based (Paszke *et al.* 2019) backend that leverages GPU processing for enhanced inference speeds; (vi) a multi-species scope, currently covering *H.sapiens*, *M.musculus*, *C.elegans*, and various mammalian species.

## 2 Materials and methods

The development of pyaging commenced with an extensive review of the literature to identify a diverse array of aging clocks, encompassing various data types, computational models, and species, as summarized in Table 1.

**Table 1.** Overview of aging clocks currently available on pyaging.^a^

Clock name	Species	Model type	Data type	Year	Citation
YingAdaptAge	*H.sapiens*	Linear	Methylation	2024	Ying *et al.* (2024)
YingDamAge	*H.sapiens*	Linear	Methylation	2024	Ying *et al.* (2024)
YingCausAge	*H.sapiens*	Linear	Methylation	2024	Ying *et al.* (2024)
DNAmFitAge	*H.sapiens*	Linear	Methylation	2023	McGreevy *et al.* (2023)
ENCen40	*H.sapiens*	Linear	Methylation	2023	Dec *et al.* (2023)
ENCen100	*H.sapiens*	Linear	Methylation	2023	Dec *et al.* (2023)
MammalianLifespan	Multi	Linear	Methylation	2023	Li *et al.* (2023)
MammalianFemale	Multi	Linear	Methylation	2023	Li *et al.* (2023)
CamilloPanHistone	*H.sapiens*	PC-ARD	Histone mark	2023	de Lima Camillo *et al.* (2023)
CamilloH3K9me3	*H.sapiens*	PC-ARD	Histone mark	2023	de Lima Camillo *et al.* (2023)
CamilloH3K9ac	*H.sapiens*	PC-ARD	Histone mark	2023	de Lima Camillo *et al.* (2023)
CamilloH3K4me3	*H.sapiens*	PC-ARD	Histone mark	2023	de Lima Camillo *et al.* (2023)
CamilloH3K4me1	*H.sapiens*	PC-ARD	Histone mark	2023	de Lima Camillo *et al.* (2023)
CamilloH3K36me3	*H.sapiens*	PC-ARD	Histone mark	2023	de Lima Camillo *et al.* (2023)
CamilloH3K27me3	*H.sapiens*	PC-ARD	Histone mark	2023	de Lima Camillo *et al.* (2023)
CamilloH3K27ac	*H.sapiens*	PC-ARD	Histone mark	2023	de Lima Camillo *et al.* (2023)
MammalianBlood3	Multi	Linear	Methylation	2023	Lu *et al.* (2023)
MammalianBlood2	Multi	Linear	Methylation	2023	Lu *et al.* (2023)
MammalianSkin3	Multi	Linear	Methylation	2023	Lu *et al.* (2023)
MammalianSkin2	Multi	Linear	Methylation	2023	Lu *et al.* (2023)
Mammalian3	Multi	Linear	Methylation	2023	Lu *et al.* (2023)
Mammalian2	Multi	Linear	Methylation	2023	Lu *et al.* (2023)
Mammalian1	Multi	Linear	Methylation	2023	Lu *et al.* (2023)
OcampoATAC2	*H.sapiens*	Linear	ATAC-seq	2023	Morandini *et al.* (2023)
OcampoATAC1	*H.sapiens*	Linear	ATAC-seq	2023	Morandini *et al.* (2023)
HRSInChPhenoAge	*H.sapiens*	Linear	Methylation	2022	Higgins-Chen *et al.* (2022)
GrimAge2	*H.sapiens*	Linear	Methylation	2022	Lu *et al.* (2022)
DunedinPACE	*H.sapiens*	Linear	Methylation	2022	Belsky *et al.* (2022)
PCSkinAndBlood	*H.sapiens*	PC-linear	Methylation	2022	Higgins-Chen *et al.* (2022)
PCPhenoAge	*H.sapiens*	PC-linear	Methylation	2022	Higgins-Chen *et al.* (2022)
PCHorvath2013	*H.sapiens*	PC-linear	Methylation	2022	Higgins-Chen *et al.* (2022)
PCHannum	*H.sapiens*	PC-linear	Methylation	2022	Higgins-Chen *et al.* (2022)
PCGrimAge	*H.sapiens*	PC-linear	Methylation	2022	Higgins-Chen *et al.* (2022)
PCDNAmTL	*H.sapiens*	PC-linear	Methylation	2022	Higgins-Chen *et al.* (2022)
AltumAge	*H.sapiens*	Neural network	Methylation	2022	de Lima Camillo *et al.* (2022)
RepliTali	*H.sapiens*	Linear	Methylation	2022	Endicott *et al.* (2022)
BiTAge	*C.elegans*	Linear	RNA-seq	2021	Meyer and Schumacher (2021)
Han	*H.sapiens*	Linear	Methylation	2020	Han *et al.* (2020)
ZhangEN	*H.sapiens*	Linear	Methylation	2019	Zhang *et al.* (2019)
ZhangBLUP	*H.sapiens*	Linear	Methylation	2019	Zhang *et al.* (2019)
GrimAge	*H.sapiens*	Linear	Methylation	2019	Lu *et al.* (2019)
DNAmTL	*H.sapiens*	Linear	Methylation	2019	Lu *et al.* (2019)
LeeControl	*H.sapiens*	Linear	Methylation	2019	Lee *et al.* (2019)
LeeRobust	*H.sapiens*	Linear	Methylation	2019	Lee *et al.* (2019)
LeeRefinedRobust	*H.sapiens*	Linear	Methylation	2019	Lee *et al.* (2019)
PedBE	*H.sapiens*	Linear	Methylation	2019	McEwen *et al.* (2020)
SkinAndBlood	*H.sapiens*	Linear	Methylation	2018	Horvath *et al.* (2018)
PhenoAge	*H.sapiens*	Linear	Methylation	2018	Levine *et al.* (2018)
DNAmPhenoAge	*H.sapiens*	Linear	Blood chemistry	2018	Levine *et al.* (2018)
Meer	*M.musculus*	Linear	Methylation	2018	Meer *et al.* (2018)
Thompson	*M.musculus*	Linear	Methylation	2018	Thompson *et al.* (2018)
Petkovich	*M.musculus*	Linear	Methylation	2017	Petkovich *et al.* (2017)
Stubbs	*M.musculus*	Linear	Methylation	2017	Stubbs *et al.* (2017)
ZhangMortality	*H.sapiens*	Linear	Methylation	2017	Zhang *et al.* (2017)
Knight	*H.sapiens*	Linear	Methylation	2016	Knight *et al.* (2016)
Lin	*H.sapiens*	Linear	Methylation	2016	Lin *et al.* (2016)
Horvath 2013	*H.sapiens*	Linear	Methylation	2013	Horvath (2013)
Hannum	*H.sapiens*	Linear	Methylation	2013	Hannum *et al.* (2013)

a More information on each biomarker is available in the documentation page at https://readthedocs.org/projects/pyaging/builds/22654195/.

Each identified model was subsequently reimplemented with a PyTorch backend to enable GPU-accelerated computations. This approach takes advantage of the fact that aging clocks, at their core, often rely on matrix multiplications, particularly within the domain of linear models, which are prevalent in aging research.

For a linear model, let β represent the vector of coefficients (including the intercept $\beta_{0}$), ϵ the vector of error terms, X the matrix of independent variables, and y the vector of dependent variable observations. The linear model can then be expressed algebraically as:

(1) $$y_{i}=\beta_{0}+\beta_{1}x_{i1}+\beta_{2}x_{i2}+\cdots +\beta_{n}x_{in}+\epsilon_{i},\;\text{for}\;i=1,\ldots ,m$$

This equation can be succinctly represented in matrix form as:

(2) y=Xβ + ϵ

where $y\in R^{m}$ is the vector of dependent variables for *m* samples, $X\in R^{m\times (n+1)}$ is the matrix of independent variables (with the first column being a vector of ones to incorporate the intercept term), $\beta \in R^{\left(n+1\right)}$ is the vector of coefficients including the intercept, and $\epsilon \in R^{m}$ is the vector of errors.

In the context of PC-based clocks, the model can be extended to incorporate dimensionality reduction via principal component analysis (PCA) before applying the linear model:

(3) $$y=(X-1\mu^{⊤})W\beta +\epsilon$$

Here, $\mu \in R^{n}$ denotes the mean vector for each independent variable, 1 is a column vector of ones of length *m* used to broadcast the mean subtraction across all samples, $W\in R^{n\times p}$ represents the rotation matrix derived from PCA, and *p* is the number of principal components retained. The term $(X-1\mu^{⊤})W$ thus represents the projection of centered data onto the principal components, upon which the linear model is applied.

This framework demonstrates that a variety of aging clocks fundamentally rely on matrix operations, making them well-suited for implementations that leverage the computational efficiencies of modern GPU architectures.

The implementation of age prediction in pyaging begins with preprocessing the data matrix. Missing values are imputed using methodologies ranging from simple mean imputation to more sophisticated techniques like KNN imputation. The input matrix is then curated to retain only the features pertinent to the selected clock, with any absent features being substituted with standardized values for the clock of interest if available or with zeros. This approach is adopted to accommodate the diversity of data types handled by pyaging, and users are duly alerted of such substitutions. Additional preprocessing steps are tailored to specific models, such as scaling for AltumAge and binarization for BiTAge. The processed data are then fed into the model for age prediction. Postprocessing steps, such as anti-log-linear transformation for certain clocks like Horvath’s (2013) and the SkinAndBlood clocks, are applied as necessary. All computations are conducted using PyTorch (Paszke *et al.* 2019) tensors within AnnData (Virshup *et al.* 2021) objects, ensuring efficient and scalable processing. The output includes the predicted values across all selected clocks, accompanied by the respective metadata, such as citations, for user reference. All of the steps are printed for clarity using a logger based on (Qiu *et al.* 2022). A simple example is as follows:

import pyaging as pya, pandas as pd

df = pd.read_pickle(’example_methylation_data.pkl’)

adata = pya.pp.df_to_adata(df, imputer_strategy=’knn’)

pya.pred.predict_age(adata,

   clock_names=[’altumage’, ’grimage2’, ’dunedinpace’])

Detailed tutorials and use-case examples are available on the documentation website: https://readthedocs.org/projects/pyaging/builds/22654195/. The code is also available on GitHub: https://github.com/rsinghlab/pyaging.

The notebook for the example analyses in this manuscript is available in the Supplementary Data. The packages used are pandas v2.1.3 (McKinney *et al.* 2011), numpy v1.26.2 (Harris *et al.* 2020), seaborn v0.12.2 (Waskom 2021), matplotlib v3.7.1 (Hunter 2007), umap-learn v0.5.5 (McInnes *et al.* 2018), scikit-learn v1.3.2 (Pedregosa *et al.* 2011), pyaging v0.1.6 (this manuscript), and biolearn v0.3.4 (Ying *et al.* 2023). The code was run on an M1 MacBook Pro.

## 3 Results

To demonstrate the capabilities of the pyaging package, I briefly analyzed 38 methylation aging clocks and biomarkers of aging using data from my previous work on AltumAge (de Lima Camillo *et al.* 2022). The dataset comprises ∼13 000 multi-tissue human samples from fetal tissue to centenarians across 142 studies, featuring beta values from overlapping probes of Illumina’s 27k, 450k, and EPIC arrays. pyaging facilitates fast and easy comparisons amongst the different models. See Supplementary Data to reproduce the figures and analyses.

**Figure 1.**
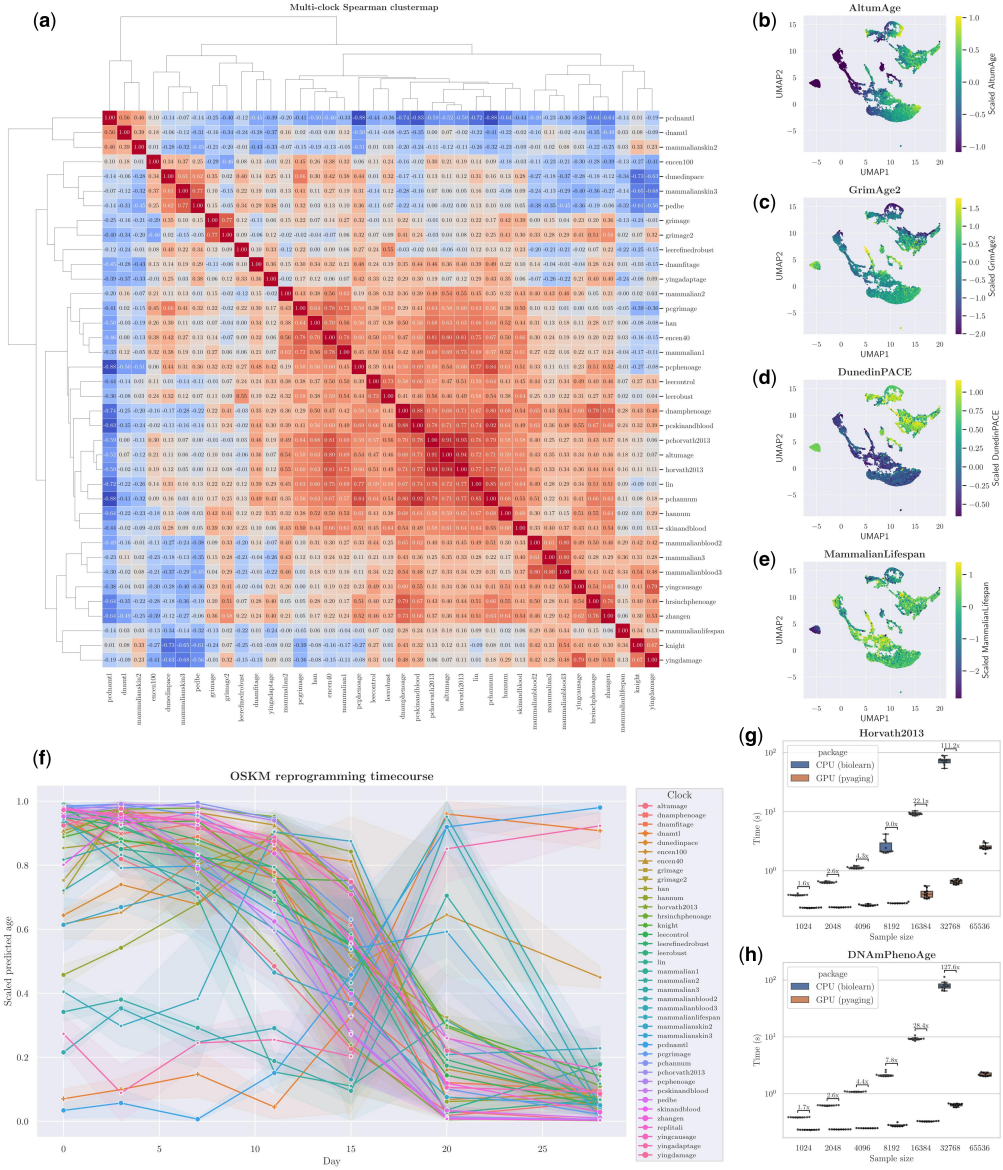
Four simple analyses with pyaging. (a) Heatmap showing Spearman’s correlation amongst 38 methylation clocks in AltumAge’s dataset. Clocks are grouped by hierarchical clustering. (b–e) UMAP plot of the top five principal components from the scaled data matrix of 38 different clocks for AltumAge’s data, highlighting AltumAge (b), GrimAge2 (c), DunedinPACE (d), and MammalianLifespan (e). (f) Line plot of 39 different clocks for the reprogramming timecourse dataset GSE54848; 95% confidence intervals are derived from 1000 bootstraps. (g, h) Performance comparison between GPU-enabled age prediction with pyaging versus CPU-only biolearn using Horvath2013 and DNAmPhenoAge. Ten random samples of size *n* from AltumAge’s data were taken to construct the boxplots. Predictions for the 65 536-sample setting for was not computed for biolearn due to memory issues.

First, with a single line of code, the output of 38 different biomarkers can be calculated. Through hierarchical clustering and Spearman correlation, expected patterns emerge (Fig. 1a). For instance, DNAmTL (Lu *et al.* 2019), and PCDNAmTL (Higgins-Chen *et al.* 2022), both estimating telomere length, cluster together. Similarly, the three human multi-tissue clocks that predict chronological age, i.e. AltumAge (de Lima Camillo *et al.* 2022), Horvath2013 (Horvath 2013), and PCHorvath2013 (Higgins-Chen *et al.* 2022), are grouped. However, there are some interesting observations. For instance, DunedinPACE (Belsky *et al.* 2022), a measure of the pace of aging, is proximate to PedBE (McEwen *et al.* 2020), an aging clock for children and adolescents. In addition, PCGrimAge (Higgins-Chen *et al.* 2022), a predictor of mortality, is near Mammalian1 (Lu *et al.* 2023), the pan-mammalian clock that predicts chronological age. Overall, pyaging makes it straightforward to contrast the performance of distinct aging clocks.

Second, given that many biomarkers are discordant, samples can be grouped into ageotypes (Ahadi *et al.* 2020). To visualize such behaviors, I ran PCA on the data matrix with the scaled result of the 38 models, followed by uniform manifold approximation and projection (UMAP) on the top five components for further dimensionality reduction (Fig. 1b–e). A chronological age predictor, a mortality predictor, and a pace of aging predictor not always agree with one another. For instance, there are islands in which AltumAge is low (Fig. 1b) but GrimAge2 (Lu *et al.* 2022) is high (Fig. 1c). Similarly, some clusters exhibit diverging patterns with DunedinPACE (Fig. 1d). Lastly, given that all samples are from human, the MammalianLifespan (Li *et al.* 2023) predicts roughly the same number for the entire data. In summary, given that different clocks measure different phenomena, pyaging makes it easy to better understand aging profiles.

Third, a burgeoning field of research within the aging research community is age reversal through epigenetic reprogramming (de Lima Camillo and Quinlan 2021, Simpson *et al.* 2021, Paine *et al.* 2023). With the expression of four transcription factors, it has been shown that the predicted age with the methylation clock Horvath2013 (Horvath 2013) is decreased to zero (Olova *et al.* 2019). To shine more light upon this process, I ran 39 clocks in a reprogramming dataset [GSE54848 (Ohnuki *et al.* 2014)]. To better compare different clocks, I scaled the data with one as the maximum and zero as the minimum of each clock (Fig. 1f). Whilst most clocks indeed show a rejuvenation event, more markedly between days 10 and 20 of reprogramming, a few such as telomere length predictors DNAmTL and PCDNAmTL increase. Others do not change meaningfully, such as the centerian predictor ENCen100 (Dec *et al.* 2023). Excitingly, some clocks such as AltumAge display a drop in predicted age at Day 3 while others such as some PC clocks only show rejuvenation at Day 11. This type of analysis can guide wet lab experiments as a tentative rejuvenation event might be missed depending on the clock used.

Fourth, one of the main advantages of the package is speed. I compared pyaging with biolearn (Ying *et al.* 2023), a preliminary CPU-based biomarker package. To compare their performance, I predicted the ages of the AltumAge data with two linear models, Horvath2013 and DNAmPhenoAge (Levine *et al.* 2018)—more complex clocks that would benefit the most from GPU acceleration, such as AltumAge, were not available on biolearn at the time of writing. I timed the line in which age is predicted for both packages given ten random samples of different sizes (Fig. 1g and f). At the lower end, pyaging displays a minor advantage with 1024 samples for the average of both clocks (0.233 versus 0.386 s). Nevertheless, the fold difference in time quickly increases with a larger sample size, with a roughly 120-fold difference with 32768 samples (0.642 versus 76.608 s). Moreover, the setting with the highest number of samples, 65536 ran out of memory with biolearn and could not be completed. While the absolute time is not substantial, given increasing data sizes and complexity of models, this will become more significant as the field develops. This becomes increasingly important as age predictors are developed for single cells given the usual large number of observations. Overall, this comparison highlights the power of GPU-acceleration enabled by pyaging.

## 4 Conclusion

Despite the abundance of aging clocks developed, a critical gap remains in integrating these diverse models for comprehensive analysis, a need only partially addressed by existing tools like methylclock and methylCYPHER. My contribution, the pyaging package, represents a significant advancement in addressing these challenges. By adopting a Python-centric approach, pyaging overcomes the limitations inherent in the R-dominated landscape of current tools, offering greater flexibility for complex models. The incorporation of a wide array of aging clocks, covering various molecular signatures, reflects the commitment to a comprehensive understanding of aging. In addition, pyaging integrates advanced modeling techniques and leverages GPU processing for enhanced computational efficiency. Its multi-species capability extends its utility across a range of gerontological studies. Overall, pyaging not only marks a substantial progress in the field of biomarkers of aging but also sets a foundation for further scientific inquiries in this rapidly developing domain.

## Supplementary Material

btae200_Supplementary_Data

## References

[btae200-B1] Ahadi S , Zhou W, Schüssler-Fiorenza Rose SM et al Personal aging markers and ageotypes revealed by deep longitudinal profiling. Nat Med 2020;26:83–90. 31932806 10.1038/s41591-019-0719-5 PMC7301912

[btae200-B2] Belsky DW , Caspi A, Corcoran DL et al Dunedinpace, a DNA methylation biomarker of the pace of aging. Elife 2022;11:e73420. 35029144 10.7554/eLife.73420 PMC8853656

[btae200-B4] de Lima Camillo LP , Quinlan RB. A ride through the epigenetic landscape: aging reversal by reprogramming. Geroscience 2021;43:463–85. 33825176 10.1007/s11357-021-00358-6 PMC8110674

[btae200-B5] de Lima Camillo LP , Lapierre LR, Singh R. A pan-tissue DNA-methylation epigenetic clock based on deep learning. NPJ Aging 2022;8:4.

[btae200-B6] de Lima Camillo LP , Asif MH, Horvath S et al Histone mark age of human tissues and cells. bioRxiv, 2023. 10.1101/2023.08.21.554165. PMC11691649 39742485

[btae200-B7] Dec E , Clement J, Cheng K et al Centenarian clocks: epigenetic clocks for validating claims of exceptional longevity. Geroscience 2023;45:1817–35. 36964402 10.1007/s11357-023-00731-7 PMC10400760

[btae200-B8] Endicott JL , Nolte PA, Shen H et al Cell division drives DNA methylation loss in late-replicating domains in primary human cells. Nat Commun 2022;13:6659. 36347867 10.1038/s41467-022-34268-8 PMC9643452

[btae200-B9] Galkin F , Mamoshina P, Kochetov K et al Deepmage: a methylation aging clock developed with deep learning. Aging Dis 2021;12:1252–62. 34341706 10.14336/AD.2020.1202 PMC8279523

[btae200-B10] Han Y , Franzen J, Stiehl T et al New targeted approaches for epigenetic age predictions. BMC Biol 2020;18:71. 32580727 10.1186/s12915-020-00807-2 PMC7315536

[btae200-B11] Hannum G , Guinney J, Zhao L et al Genome-wide methylation profiles reveal quantitative views of human aging rates. Mol Cell 2013;49:359–67. 23177740 10.1016/j.molcel.2012.10.016 PMC3780611

[btae200-B12] Harris CR , Millman KJ, van der Walt SJ et al Array programming with numpy. Nature 2020;585:357–62. 32939066 10.1038/s41586-020-2649-2 PMC7759461

[btae200-B13] Higgins-Chen AT , Thrush KL, Wang Y et al A computational solution for bolstering reliability of epigenetic clocks: implications for clinical trials and longitudinal tracking. Nat Aging 2022;2:644–61. 36277076 10.1038/s43587-022-00248-2 PMC9586209

[btae200-B14] Horvath S. DNA methylation age of human tissues and cell types. Genome Biol 2013;14:R115–20. 24138928 10.1186/gb-2013-14-10-r115 PMC4015143

[btae200-B15] Horvath S , Oshima J, Martin GM et al Epigenetic clock for skin and blood cells applied to Hutchinson Gilford progeria syndrome and ex vivo studies. Aging (Albany NY) 2018;10:1758–75. 30048243 10.18632/aging.101508 PMC6075434

[btae200-B16] Hunter JD. Matplotlib: a 2D graphics environment. Comput Sci Eng 2007;9:90–5.

[btae200-B17] Knight AK , Craig JM, Theda C et al An epigenetic clock for gestational age at birth based on blood methylation data. Genome Biol 2016;17:206–11. 27717399 10.1186/s13059-016-1068-z PMC5054584

[btae200-B18] Lee Y , Choufani S, Weksberg R et al Placental epigenetic clocks: estimating gestational age using placental DNA methylation levels. Aging (Albany NY) 2019;11:4238–53. 31235674 10.18632/aging.102049 PMC6628997

[btae200-B19] Levine ME , Lu AT, Quach A et al An epigenetic biomarker of aging for lifespan and healthspan. Aging (Albany NY) 2018;10:573–91. 29676998 10.18632/aging.101414 PMC5940111

[btae200-B20] Li CZ , Haghani A, Yan Q et al Epigenetic predictors of species maximum lifespan and other life history traits in mammals. bioRxiv, 2023. 10.1101/2023.11.02.565286. PMC11160467 38848365

[btae200-B21] Lin Q , Weidner CI, Costa IG et al DNA methylation levels at individual age-associated CPG sites can be indicative for life expectancy. Aging (Albany, NY) 2016;8:394–401. 26928272 10.18632/aging.100908 PMC4789590

[btae200-B22] Lu AT , Fei Z, Haghani A et al Universal DNA methylation age across mammalian tissues. Nat Aging 2023;3:1144–66. 37563227 10.1038/s43587-023-00462-6 PMC10501909

[btae200-B23] Lu AT , Quach A, Wilson JG et al DNA methylation grimage strongly predicts lifespan and healthspan. Aging (Albany, NY) 2019;11:303–27. 30669119 10.18632/aging.101684 PMC6366976

[btae200-B24] Lu AT , Binder AM, Zhang J et al DNA methylation grimage version 2. Aging (Albany NY) 2022;14:9484. 36516495 10.18632/aging.204434 PMC9792204

[btae200-B25] MacKay DJ. Information Theory, Inference and Learning Algorithms. Cambridge, UK: Cambridge University Press, 2003.

[btae200-B26] McEwen LM , O'Donnell KJ, McGill MG et al The PEDBE clock accurately estimates DNA methylation age in pediatric buccal cells. Proc Natl Acad Sci USA 2020;117:23329–35. 31611402 10.1073/pnas.1820843116 PMC7519312

[btae200-B27] McGreevy KM , Radak Z, Torma F et al DNAmfitage: biological age indicator incorporating physical fitness. Aging (Albany, NY) 2023;15:3904–38. 36812475 10.18632/aging.204538 PMC10258016

[btae200-B28] McInnes L , Healy J, Saul N et al Umap: uniform manifold approximation and projection. JOSS 2018;3:861.

[btae200-B29] McKinney W et al pandas: a foundational python library for data analysis and statistics. Python High Performance Sci Comput 2011;14:1–9.

[btae200-B30] Meer MV , Podolskiy DI, Tyshkovskiy A et al A whole lifespan mouse multi-tissue DNA methylation clock. Elife 2018;7:e40675. 30427307 10.7554/eLife.40675 PMC6287945

[btae200-B31] Meyer DH , Schumacher B. Bit age: a transcriptome-based aging clock near the theoretical limit of accuracy. Aging Cell 2021;20:e13320. 33656257 10.1111/acel.13320 PMC7963339

[btae200-B32] Moqri M , Herzog C, Poganik JR et al; Biomarkers of Aging Consortium. Biomarkers of aging for the identification and evaluation of longevity interventions. Cell 2023;186:3758–75. 37657418 10.1016/j.cell.2023.08.003 PMC11088934

[btae200-B33] Morandini F , Rechsteiner C, Perez K et al ATAC-clock: an aging clock based on chromatin accessibility. GeroScience 2024;46:1789–1806. 10.1007/s11357-023-00986-0. 37924441 PMC10828344

[btae200-B34] Ohnuki M , Tanabe K, Sutou K et al Dynamic regulation of human endogenous retroviruses mediates factor-induced reprogramming and differentiation potential. Proc Natl Acad Sci USA 2014;111:12426–31. 25097266 10.1073/pnas.1413299111 PMC4151758

[btae200-B35] Olova N , Simpson DJ, Marioni RE et al Partial reprogramming induces a steady decline in epigenetic age before loss of somatic identity. Aging Cell 2019;18:e12877. 30450724 10.1111/acel.12877 PMC6351826

[btae200-B36] Paine PT , Nguyen A, Ocampo A. Partial cellular reprogramming: a deep dive into an emerging rejuvenation technology. Aging Cell 2023;23:e14039. page 38040663 10.1111/acel.14039 PMC10861195

[btae200-B37] Paszke A , Gross S, Massa F et al Pytorch: an imperative style, high-performance deep learning library. Adv Neural Inf Process Syst 2019;32.

[btae200-B38] Pedregosa F , Varoquaux G, Gramfort A et al Scikit-learn: machine learning in Python. J Mach Learn Res 2011;12:2825–30.

[btae200-B39] Pelegí-Sisó D , de Prado P, Ronkainen J et al methylclock: a bioconductor package to estimate DNA methylation age. Bioinformatics 2021;37:1759–60. 32960939 10.1093/bioinformatics/btaa825

[btae200-B40] Petkovich DA , Podolskiy DI, Lobanov AV et al Using DNA methylation profiling to evaluate biological age and longevity interventions. Cell Metab 2017;25:954–60.e6. 28380383 10.1016/j.cmet.2017.03.016 PMC5578459

[btae200-B41] Qiu X , Zhang Y, Martin-Rufino JD et al Mapping transcriptomic vector fields of single cells. Cell 2022;185:690–711.e45. 35108499 10.1016/j.cell.2021.12.045 PMC9332140

[btae200-B42] Simpson DJ , Olova NN, Chandra T. Cellular reprogramming and epigenetic rejuvenation. Clin Epigenet 2021;13:170–10. 10.1186/s13148-021-01158-7 PMC8419998 34488874

[btae200-B43] Stubbs TM , Bonder MJ, Stark A-K et al; BI Ageing Clock Team. Multi-tissue DNA methylation age predictor in mouse. Genome Biol 2017;18:68–14. 28399939 10.1186/s13059-017-1203-5 PMC5389178

[btae200-B44] Thompson MJ , Chwiałkowska K, Rubbi L et al A multi-tissue full lifespan epigenetic clock for mice. Aging (Albany, NY) 2018;10:2832–54. 30348905 10.18632/aging.101590 PMC6224226

[btae200-B45] Thrush KL , Higgins-Chen AT, Liu Z et al R methylcipher: a methylation clock investigational package for hypothesis-driven evaluation & research. bioRxiv, 2022. 10.1101/2022.07.13.499978.

[btae200-B4700] Virshup I , Sergei R, Theis FJ et al anndata: Annotated data. *BioRxiv* 2021:2021–12. 10.1101/2021.12.16.473007.

[btae200-B46] Waskom ML. Seaborn: statistical data visualization. JOSS 2021;6:3021.

[btae200-B47] Ying K , Paulson S, Perez-Guevara M et al Biolearn, an open-source library for biomarkers of aging. bioRxiv, 2023. 10.1101/2023.12.02.569722.

[btae200-B48] Ying K , Liu H, Tarkhov AE et al Causality-enriched epigenetic age uncouples damage and adaptation. Nat Aging 2024;4:231–46. 38243142 10.1038/s43587-023-00557-0 PMC11070280

[btae200-B49] Zhang Q , Vallerga CL, Walker RM et al Improved precision of epigenetic clock estimates across tissues and its implication for biological ageing. Genome Med 2019;11:54–11. 31443728 10.1186/s13073-019-0667-1 PMC6708158

[btae200-B50] Zhang Y , Wilson R, Heiss J et al DNA methylation signatures in peripheral blood strongly predict all-cause mortality. Nat Commun 2017;8:14617. 28303888 10.1038/ncomms14617 PMC5357865

