# Supplementary material for: pyaging: a Python-based compendium of GPU-optimized aging clocks
Source: Bioinformatics. 2024 Apr 11;40(4):btae200. doi: 10.1093/bioinformatics/btae200 (PMC11058068; doi:10.1093/bioinformatics/btae200)
Supplement: btae200_Supplementary_Data [file btae200_supplementary_data.zip › supplementary.html]

pyaging\_manuscript


# Supplementary Material¶

First, let's import the different packages necessary for the analyses contained in the manuscript and set some settings for the plots.

In [1]:

```
import pandas as pd # 2.1.3
import pyaging as pya # 0.1.6
import numpy as np # 1.26.2
import time 
from tqdm import tqdm # 4.65.0
from biolearn.model_gallery import ModelGallery # 0.3.4
from biolearn.data_library import DataLibrary # 0.3.4
import seaborn as sns # 0.12.2
import matplotlib # 3.7.1
import matplotlib.pyplot as plt # 1.11.1
from scipy.stats import spearmanr
import gc
import umap # 0.5.5
from sklearn.decomposition import PCA # 1.3.2
from sklearn.preprocessing import RobustScaler # 1.3.2
import GEOparse # 2.0.3
np.random.seed(42)
matplotlib.rcParams['text.usetex'] = True
matplotlib.rcParams['font.family'] = 'serif'
matplotlib.rcParams['font.serif'] = 'Times New Roman'
matplotlib.rcParams['font.size'] = 12
```

```
/Users/lucascamillo/mambaforge/envs/kaggle_icr/lib/python3.11/site-packages/tqdm/auto.py:21: TqdmWarning: IProgress not found. Please update jupyter and ipywidgets. See https://ipywidgets.readthedocs.io/en/stable/user_install.html
  from .autonotebook import tqdm as notebook_tqdm
```

# Experiment 1: GPU vs CPU performance¶

Let's compare the performance of simple linear models, which are available both in pyaging and biolearn, in samples of different sizes. In this experiment, let's randomly draw samples of different sizes from the data that was used to train AltumAge (https://www.nature.com/articles/s41514-022-00085-y).

In [49]:

```
df = pd.read_pickle('example_data/altumage_data.pkl')
```

## GPU (pyaging) time experiment¶

In [ ]:

```
sample_sizes = 2**np.arange(7, 17, 1)
clocks = ['Horvath2013', 'DNAmPhenoAge']

t = []
n = []
c = []
np.random.seed(42)
for clock in clocks:
    for sample_size in tqdm(sample_sizes):
        for _ in range(10):
            sample_indices = np.random.randint(0, len(df), sample_size)
            df_subset = df.iloc[sample_indices,:]
            df_subset.index = np.arange(0, len(df_subset), 1)
            adata = pya.pp.df_to_adata(df_subset, imputer_strategy='mean', verbose=False)
            start = time.time()
            pya.pred.predict_age(adata, clocks, batch_size=2**14, verbose=False)
            end = time.time()
            time_passed = end - start
            print(time_passed)
            t += [time_passed]
            n += [sample_size]
            c += [clock]

results = pd.DataFrame(np.array([t, n, c]).T, columns=['time', 'sample_size', 'clock'])
results['time'] = results['time'].astype(float)
results['sample_size'] = results['sample_size'].astype(int)
results.to_pickle('example_data/pyaging_results.pkl')
```

## CPU (biolearn) time experiment¶

For biolearn, the condition with 65536 (2 to the power of 16) samples was removed because the kernel fails every time.

In [ ]:

```
sample_sizes = 2**np.arange(7, 16, 1) # With 2**17 the kernel dies 
clocks = ['Horvathv1', 'PhenoAge']

gallery = ModelGallery()
data_source = DataLibrary().get("GSE41169") # This is just to create a Data object to add the DNAm data later
data=data_source.load()

t = []
n = []
c = []
np.random.seed(42)
for clock in clocks:
    for sample_size in tqdm(sample_sizes):
        for _ in range(10):
            sample_indices = np.random.randint(0, len(df), sample_size)
            df_subset = df.iloc[sample_indices,:]
            df_subset.index = np.arange(0, len(df_subset), 1)
            data.dnam = df_subset.T
            start = time.time()
            horvath_results = gallery.get(clock).predict(data)
            end = time.time()
            time_passed = end - start
            t += [time_passed]
            n += [sample_size]
            c += [clock]
            
results = pd.DataFrame(np.array([t, n, c]).T, columns=['time', 'sample_size', 'clock'])
results['time'] = results['time'].astype(float)
results['sample_size'] = results['sample_size'].astype(int)
results.to_pickle('example_data/biolearn_results.pkl')
```

## Boxplot plot¶

In [43]:

```
results_biolearn = pd.read_pickle('example_data/biolearn_results.pkl')
results_biolearn['package'] = 'CPU (biolearn)'
results_pyaging = pd.read_pickle('example_data/pyaging_results.pkl')
results_pyaging['package'] = 'GPU (pyaging)'
results = pd.concat([results_biolearn, results_pyaging])
```

In [44]:

```
# Set the aesthetic style of the plots
sns.set(style="whitegrid")  # or "whitegrid" for a lighter grey

matplotlib.rcParams['text.usetex'] = True
matplotlib.rcParams['font.family'] = 'serif'
matplotlib.rcParams['font.serif'] = 'Times New Roman'
matplotlib.rcParams['font.size'] = 12

# Increase the figure size and create subplots
fig, axes = plt.subplots(2, 1, figsize=(5, 8.5), dpi=300)

# Data filtering for the first plot
data_horvath = results[['horvath' in clock.lower() for clock in results.clock]]

# Create the first boxplot
ax1 = sns.boxplot(data=data_horvath, x='sample_size', y='time', hue='package', showfliers=False, ax=axes[0])

# Overlay individual points
sns.swarmplot(data=data_horvath, x='sample_size', y='time', hue='package', color='black', size=3, dodge=True, ax=axes[0], alpha=0.8, legend=False)

# Group the data by sample size and package, then calculate the median time for each group
grouped_mean = data_horvath.groupby(['sample_size', 'package'])['time'].mean().reset_index()
grouped_max = data_horvath.groupby(['sample_size', 'package'])['time'].max().reset_index()
packages = grouped_mean['package'].unique()
for i, sample_size in enumerate(grouped_mean['sample_size'].unique()):
    sample_data_mean = grouped_mean[(grouped_mean['sample_size'] == sample_size) & (grouped_mean['package'].isin(packages))]
    sample_data_max = grouped_max[(grouped_max['sample_size'] == sample_size) & (grouped_max['package'].isin(packages))]
    if len(sample_data_mean) == 2:
        # Calculate the positions for the brackets
        x1, x2 = i - 0.2, i + 0.2  # Adjust these values as needed to position the brackets correctly
        y, h = max(sample_data_max['time']) * 1.2, max(sample_data_max['time']) * 0.1  # y and height of the bracket
        
        # Draw the first line (left vertical)
        ax1.plot([x1, x1], [y, y+h], lw=1, color="black")
        # Draw the top line
        ax1.plot([x1, x2], [y+h, y+h], lw=1, color="black")
        # Draw the second line (right vertical)
        ax1.plot([x2, x2], [y, y+h], lw=1, color="black")
        
        # Calculate the ratio
        ratio = sample_data_mean['time'].iloc[0] / sample_data_mean['time'].iloc[1]
        # Annotate the bracket with the ratio
        ax1.text((x1+x2)*0.5, y+h, f'{ratio:.1f}x', ha='center', va='bottom', fontsize=9)

# Set the title and labels for the first plot
ax1.set_title(r'\textbf{Horvath2013}', fontsize=14)
ax1.set_xlabel('Sample size', fontsize=12, weight='bold')
ax1.set_ylabel('Time (s)', fontsize=12, weight='bold')
ax1.legend(title='package', title_fontsize='12', loc='upper left', fontsize='12')
ax1.set_yscale('log')

# Data filtering for the second plot
data_not_horvath = results[['horvath' not in clock.lower() for clock in results.clock]]

# Create the second boxplot
ax2 = sns.boxplot(data=data_not_horvath, x='sample_size', y='time', hue='package', showfliers=False, ax=axes[1])

# Overlay individual points
sns.swarmplot(data=data_not_horvath, x='sample_size', y='time', hue='package', color='black', size=3, dodge=True, ax=axes[1], alpha=0.8, legend=False)

# Group the data by sample size and package, then calculate the median time for each group
grouped_mean = data_not_horvath.groupby(['sample_size', 'package'])['time'].mean().reset_index()
grouped_max = data_not_horvath.groupby(['sample_size', 'package'])['time'].max().reset_index()
packages = grouped_mean['package'].unique()
for i, sample_size in enumerate(grouped_mean['sample_size'].unique()):
    sample_data_mean = grouped_mean[(grouped_mean['sample_size'] == sample_size) & (grouped_mean['package'].isin(packages))]
    sample_data_max = grouped_max[(grouped_max['sample_size'] == sample_size) & (grouped_max['package'].isin(packages))]
    if len(sample_data_mean) == 2:
        # Calculate the positions for the brackets
        x1, x2 = i - 0.2, i + 0.2  # Adjust these values as needed to position the brackets correctly
        y, h = max(sample_data_max['time']) * 1.2, max(sample_data_max['time']) * 0.1  # y and height of the bracket
        
        # Draw the first line (left vertical)
        ax2.plot([x1, x1], [y, y+h], lw=1, color="black")
        # Draw the top line
        ax2.plot([x1, x2], [y+h, y+h], lw=1, color="black")
        # Draw the second line (right vertical)
        ax2.plot([x2, x2], [y, y+h], lw=1, color="black")
        
        # Calculate the ratio
        ratio = sample_data_mean['time'].iloc[0] / sample_data_mean['time'].iloc[1]
        # Annotate the bracket with the ratio
        ax2.text((x1+x2)*0.5, y+h, f'{ratio:.1f}x', ha='center', va='bottom', fontsize=9)

# Set the title and labels for the second plot
ax2.set_title(r'\textbf{DNAmPhenoAge}', fontsize=14)
ax2.set_xlabel('Sample size', fontsize=12, weight='bold')
ax2.set_ylabel('Time (s)', fontsize=12, weight='bold')
ax2.legend(title='package', title_fontsize='12', loc='upper left', fontsize='12')
ax2.set_yscale('log')

# Adjust the layout
plt.tight_layout()

# Save the figure with high resolution
plt.savefig('example_data/figure1b.pdf', dpi=300, bbox_inches='tight')

plt.show()
```

```
/Users/lucascamillo/mambaforge/envs/kaggle_icr/lib/python3.11/site-packages/seaborn/_oldcore.py:1498: FutureWarning: is_categorical_dtype is deprecated and will be removed in a future version. Use isinstance(dtype, CategoricalDtype) instead
  if pd.api.types.is_categorical_dtype(vector):
/Users/lucascamillo/mambaforge/envs/kaggle_icr/lib/python3.11/site-packages/seaborn/_oldcore.py:1498: FutureWarning: is_categorical_dtype is deprecated and will be removed in a future version. Use isinstance(dtype, CategoricalDtype) instead
  if pd.api.types.is_categorical_dtype(vector):
/Users/lucascamillo/mambaforge/envs/kaggle_icr/lib/python3.11/site-packages/seaborn/_oldcore.py:1498: FutureWarning: is_categorical_dtype is deprecated and will be removed in a future version. Use isinstance(dtype, CategoricalDtype) instead
  if pd.api.types.is_categorical_dtype(vector):
/Users/lucascamillo/mambaforge/envs/kaggle_icr/lib/python3.11/site-packages/seaborn/_oldcore.py:1498: FutureWarning: is_categorical_dtype is deprecated and will be removed in a future version. Use isinstance(dtype, CategoricalDtype) instead
  if pd.api.types.is_categorical_dtype(vector):
/Users/lucascamillo/mambaforge/envs/kaggle_icr/lib/python3.11/site-packages/seaborn/_oldcore.py:1498: FutureWarning: is_categorical_dtype is deprecated and will be removed in a future version. Use isinstance(dtype, CategoricalDtype) instead
  if pd.api.types.is_categorical_dtype(vector):
/Users/lucascamillo/mambaforge/envs/kaggle_icr/lib/python3.11/site-packages/seaborn/_oldcore.py:1498: FutureWarning: is_categorical_dtype is deprecated and will be removed in a future version. Use isinstance(dtype, CategoricalDtype) instead
  if pd.api.types.is_categorical_dtype(vector):
/Users/lucascamillo/mambaforge/envs/kaggle_icr/lib/python3.11/site-packages/seaborn/_oldcore.py:1498: FutureWarning: is_categorical_dtype is deprecated and will be removed in a future version. Use isinstance(dtype, CategoricalDtype) instead
  if pd.api.types.is_categorical_dtype(vector):
/Users/lucascamillo/mambaforge/envs/kaggle_icr/lib/python3.11/site-packages/seaborn/_oldcore.py:1498: FutureWarning: is_categorical_dtype is deprecated and will be removed in a future version. Use isinstance(dtype, CategoricalDtype) instead
  if pd.api.types.is_categorical_dtype(vector):
/Users/lucascamillo/mambaforge/envs/kaggle_icr/lib/python3.11/site-packages/seaborn/_oldcore.py:1498: FutureWarning: is_categorical_dtype is deprecated and will be removed in a future version. Use isinstance(dtype, CategoricalDtype) instead
  if pd.api.types.is_categorical_dtype(vector):
/Users/lucascamillo/mambaforge/envs/kaggle_icr/lib/python3.11/site-packages/seaborn/_oldcore.py:1498: FutureWarning: is_categorical_dtype is deprecated and will be removed in a future version. Use isinstance(dtype, CategoricalDtype) instead
  if pd.api.types.is_categorical_dtype(vector):
/Users/lucascamillo/mambaforge/envs/kaggle_icr/lib/python3.11/site-packages/seaborn/_oldcore.py:1498: FutureWarning: is_categorical_dtype is deprecated and will be removed in a future version. Use isinstance(dtype, CategoricalDtype) instead
  if pd.api.types.is_categorical_dtype(vector):
/Users/lucascamillo/mambaforge/envs/kaggle_icr/lib/python3.11/site-packages/seaborn/_oldcore.py:1498: FutureWarning: is_categorical_dtype is deprecated and will be removed in a future version. Use isinstance(dtype, CategoricalDtype) instead
  if pd.api.types.is_categorical_dtype(vector):
/Users/lucascamillo/mambaforge/envs/kaggle_icr/lib/python3.11/site-packages/seaborn/_oldcore.py:1498: FutureWarning: is_categorical_dtype is deprecated and will be removed in a future version. Use isinstance(dtype, CategoricalDtype) instead
  if pd.api.types.is_categorical_dtype(vector):
/Users/lucascamillo/mambaforge/envs/kaggle_icr/lib/python3.11/site-packages/seaborn/categorical.py:166: FutureWarning: Setting a gradient palette using color= is deprecated and will be removed in version 0.13. Set `palette='dark:black'` for same effect.
  warnings.warn(msg, FutureWarning)
/Users/lucascamillo/mambaforge/envs/kaggle_icr/lib/python3.11/site-packages/seaborn/_oldcore.py:1498: FutureWarning: is_categorical_dtype is deprecated and will be removed in a future version. Use isinstance(dtype, CategoricalDtype) instead
  if pd.api.types.is_categorical_dtype(vector):
/Users/lucascamillo/mambaforge/envs/kaggle_icr/lib/python3.11/site-packages/seaborn/_oldcore.py:1119: FutureWarning: use_inf_as_na option is deprecated and will be removed in a future version. Convert inf values to NaN before operating instead.
  with pd.option_context('mode.use_inf_as_na', True):
/Users/lucascamillo/mambaforge/envs/kaggle_icr/lib/python3.11/site-packages/seaborn/_oldcore.py:1119: FutureWarning: use_inf_as_na option is deprecated and will be removed in a future version. Convert inf values to NaN before operating instead.
  with pd.option_context('mode.use_inf_as_na', True):
/Users/lucascamillo/mambaforge/envs/kaggle_icr/lib/python3.11/site-packages/seaborn/categorical.py:3544: UserWarning: 10.0% of the points cannot be placed; you may want to decrease the size of the markers or use stripplot.
  warnings.warn(msg, UserWarning)
/Users/lucascamillo/mambaforge/envs/kaggle_icr/lib/python3.11/site-packages/seaborn/_oldcore.py:1498: FutureWarning: is_categorical_dtype is deprecated and will be removed in a future version. Use isinstance(dtype, CategoricalDtype) instead
  if pd.api.types.is_categorical_dtype(vector):
/Users/lucascamillo/mambaforge/envs/kaggle_icr/lib/python3.11/site-packages/seaborn/_oldcore.py:1498: FutureWarning: is_categorical_dtype is deprecated and will be removed in a future version. Use isinstance(dtype, CategoricalDtype) instead
  if pd.api.types.is_categorical_dtype(vector):
/Users/lucascamillo/mambaforge/envs/kaggle_icr/lib/python3.11/site-packages/seaborn/_oldcore.py:1498: FutureWarning: is_categorical_dtype is deprecated and will be removed in a future version. Use isinstance(dtype, CategoricalDtype) instead
  if pd.api.types.is_categorical_dtype(vector):
/Users/lucascamillo/mambaforge/envs/kaggle_icr/lib/python3.11/site-packages/seaborn/_oldcore.py:1498: FutureWarning: is_categorical_dtype is deprecated and will be removed in a future version. Use isinstance(dtype, CategoricalDtype) instead
  if pd.api.types.is_categorical_dtype(vector):
/Users/lucascamillo/mambaforge/envs/kaggle_icr/lib/python3.11/site-packages/seaborn/_oldcore.py:1498: FutureWarning: is_categorical_dtype is deprecated and will be removed in a future version. Use isinstance(dtype, CategoricalDtype) instead
  if pd.api.types.is_categorical_dtype(vector):
/Users/lucascamillo/mambaforge/envs/kaggle_icr/lib/python3.11/site-packages/seaborn/_oldcore.py:1498: FutureWarning: is_categorical_dtype is deprecated and will be removed in a future version. Use isinstance(dtype, CategoricalDtype) instead
  if pd.api.types.is_categorical_dtype(vector):
/Users/lucascamillo/mambaforge/envs/kaggle_icr/lib/python3.11/site-packages/seaborn/_oldcore.py:1498: FutureWarning: is_categorical_dtype is deprecated and will be removed in a future version. Use isinstance(dtype, CategoricalDtype) instead
  if pd.api.types.is_categorical_dtype(vector):
/Users/lucascamillo/mambaforge/envs/kaggle_icr/lib/python3.11/site-packages/seaborn/_oldcore.py:1498: FutureWarning: is_categorical_dtype is deprecated and will be removed in a future version. Use isinstance(dtype, CategoricalDtype) instead
  if pd.api.types.is_categorical_dtype(vector):
/Users/lucascamillo/mambaforge/envs/kaggle_icr/lib/python3.11/site-packages/seaborn/_oldcore.py:1498: FutureWarning: is_categorical_dtype is deprecated and will be removed in a future version. Use isinstance(dtype, CategoricalDtype) instead
  if pd.api.types.is_categorical_dtype(vector):
/Users/lucascamillo/mambaforge/envs/kaggle_icr/lib/python3.11/site-packages/seaborn/_oldcore.py:1498: FutureWarning: is_categorical_dtype is deprecated and will be removed in a future version. Use isinstance(dtype, CategoricalDtype) instead
  if pd.api.types.is_categorical_dtype(vector):
/Users/lucascamillo/mambaforge/envs/kaggle_icr/lib/python3.11/site-packages/seaborn/_oldcore.py:1498: FutureWarning: is_categorical_dtype is deprecated and will be removed in a future version. Use isinstance(dtype, CategoricalDtype) instead
  if pd.api.types.is_categorical_dtype(vector):
/Users/lucascamillo/mambaforge/envs/kaggle_icr/lib/python3.11/site-packages/seaborn/_oldcore.py:1498: FutureWarning: is_categorical_dtype is deprecated and will be removed in a future version. Use isinstance(dtype, CategoricalDtype) instead
  if pd.api.types.is_categorical_dtype(vector):
/Users/lucascamillo/mambaforge/envs/kaggle_icr/lib/python3.11/site-packages/seaborn/_oldcore.py:1498: FutureWarning: is_categorical_dtype is deprecated and will be removed in a future version. Use isinstance(dtype, CategoricalDtype) instead
  if pd.api.types.is_categorical_dtype(vector):
/Users/lucascamillo/mambaforge/envs/kaggle_icr/lib/python3.11/site-packages/seaborn/categorical.py:166: FutureWarning: Setting a gradient palette using color= is deprecated and will be removed in version 0.13. Set `palette='dark:black'` for same effect.
  warnings.warn(msg, FutureWarning)
/Users/lucascamillo/mambaforge/envs/kaggle_icr/lib/python3.11/site-packages/seaborn/_oldcore.py:1498: FutureWarning: is_categorical_dtype is deprecated and will be removed in a future version. Use isinstance(dtype, CategoricalDtype) instead
  if pd.api.types.is_categorical_dtype(vector):
/Users/lucascamillo/mambaforge/envs/kaggle_icr/lib/python3.11/site-packages/seaborn/_oldcore.py:1119: FutureWarning: use_inf_as_na option is deprecated and will be removed in a future version. Convert inf values to NaN before operating instead.
  with pd.option_context('mode.use_inf_as_na', True):
/Users/lucascamillo/mambaforge/envs/kaggle_icr/lib/python3.11/site-packages/seaborn/_oldcore.py:1119: FutureWarning: use_inf_as_na option is deprecated and will be removed in a future version. Convert inf values to NaN before operating instead.
  with pd.option_context('mode.use_inf_as_na', True):
/Users/lucascamillo/mambaforge/envs/kaggle_icr/lib/python3.11/site-packages/seaborn/categorical.py:3544: UserWarning: 10.0% of the points cannot be placed; you may want to decrease the size of the markers or use stripplot.
  warnings.warn(msg, UserWarning)
/Users/lucascamillo/mambaforge/envs/kaggle_icr/lib/python3.11/site-packages/seaborn/categorical.py:3544: UserWarning: 10.0% of the points cannot be placed; you may want to decrease the size of the markers or use stripplot.
  warnings.warn(msg, UserWarning)
```

## Results table¶

In [129]:

```
results.groupby(['package', 'clock', 'sample_size',]).mean()
```

Out[129]:

|  |  |  | time |
| --- | --- | --- | --- |
| package | clock | sample\_size |  |
| CPU (biolearn) | Horvathv1 | 1024 | 0.388577 |
|  |  | 2048 | 0.635240 |
|  |  | 4096 | 1.123038 |
|  |  | 8192 | 2.556346 |
|  |  | 16384 | 9.237364 |
|  |  | 32768 | 72.320359 |
|  | PhenoAge | 1024 | 0.383827 |
|  |  | 2048 | 0.619965 |
|  |  | 4096 | 1.080929 |
|  |  | 8192 | 2.152665 |
|  |  | 16384 | 9.267073 |
|  |  | 32768 | 80.896522 |
| GPU (pyaging) | DNAmPhenoAge | 1024 | 0.231943 |
|  |  | 2048 | 0.235411 |
|  |  | 4096 | 0.247016 |
|  |  | 8192 | 0.277214 |
|  |  | 16384 | 0.326074 |
|  |  | 32768 | 0.633882 |
|  |  | 65536 | 2.172954 |
|  | Horvath2013 | 1024 | 0.235701 |
|  |  | 2048 | 0.240263 |
|  |  | 4096 | 0.260377 |
|  |  | 8192 | 0.283134 |
|  |  | 16384 | 0.417155 |
|  |  | 32768 | 0.650480 |
|  |  | 65536 | 2.523426 |

# Experiment 2: correlations amongst several different clocks¶

One way to determine which clocks are more similar to others for a general analysis is to check for correlations. Therefore, using the same data used for the benchmarking experiments previously, let's compare the predicted age of different models.

In [25]:

```
df = pd.read_pickle('example_data/altumage_data.pkl')
df['Homo sapiens'] = 1
adata = pya.pp.df_to_adata(df, imputer_strategy='mean', verbose=False)

dnam_clocks = [
    'altumage',
    'dnamphenoage',
    'dnamfitage',
    'dnamtl',
    'dunedinpace',
    'encen100',
    'encen40',
    'grimage',
    'grimage2',
    'han',
    'hannum',
    'horvath2013',
    'hrsinchphenoage',
    'knight',
    'leecontrol',
    'leerefinedrobust',
    'leerobust',
    'lin',
    'mammalian1',
    'mammalian2',
    'mammalian3',
    'mammalianblood2',
    'mammalianblood3',
    'mammalianlifespan',
    'mammalianskin2',
    'mammalianskin3',
    'pcdnamtl',
    'pcgrimage',
    'pchannum',
    'pchorvath2013',
    'pcphenoage',
    'pcskinandblood',
    'pedbe',
    'skinandblood',
    'zhangen',
    'yingcausage',
    'yingadaptage',
    'yingdamage',
]
pya.pred.predict_age(adata, dnam_clocks, verbose=False)
    
adata.obs.to_pickle('example_data/altumage_data_results.pkl')
```

## UMAP plot¶

In [32]:

```
sns.set(style="darkgrid")  # or "whitegrid" for a lighter grey

np.random.seed(1)

altumage_data_results = pd.read_pickle('example_data/altumage_data_results.pkl')

# Standardize the data
scaler = RobustScaler()
df_scaled = pd.DataFrame(scaler.fit_transform(altumage_data_results), columns=altumage_data_results.columns)

# Perform PCA
pca = PCA(n_components=5)  # choose a number of components
principal_components = pca.fit_transform(df_scaled)

# Perform UMAP on PCA results
reducer = umap.UMAP()
embedding = reducer.fit_transform(principal_components)

# Convert to a DataFrame for plotting
umap_df = pd.DataFrame(data=embedding, columns=['UMAP1', 'UMAP2'])

# Plotting three UMAP plots side by side
fig, axes = plt.subplots(4, 1, figsize=(4.5, 14))

# List of columns to use for coloring each plot
color_columns = ['AltumAge', 'GrimAge2', 'DunedinPACE', 'MammalianLifespan']

for ax, column in zip(axes.flatten(), color_columns):
    color_values = df_scaled[column.lower()]
    vmin, vmax = np.percentile(color_values, [1, 99])
    scatter = ax.scatter(umap_df['UMAP1'], umap_df['UMAP2'], c=color_values, alpha=0.9, s=1, cmap='viridis', vmin=vmin, vmax=vmax)
    fig.colorbar(scatter, ax=ax, label=f'Scaled {column}')
    ax.set_title(r'$\bf{' + f'{column}' + '}$', fontsize=14)
    ax.set_xlabel('UMAP1', fontsize=12)
    ax.set_ylabel('UMAP2', fontsize=12)
    ax.grid(True, linestyle='--', alpha=0.7)
    ax.tick_params(axis='both', which='major', labelsize=10)
    ax.spines['top'].set_visible(False)
    ax.spines['right'].set_visible(False)

plt.tight_layout()
plt.savefig('example_data/figure1c.pdf', dpi=300, bbox_inches='tight')
plt.show()
```

## Clustermap plot¶

In [41]:

```
matplotlib.rcParams['text.usetex'] = True
matplotlib.rcParams['font.family'] = 'serif'
matplotlib.rcParams['font.serif'] = 'Times New Roman'
matplotlib.rcParams['font.size'] = 12
```

In [42]:

```
corr_matrix = altumage_data_results.corr('spearman')

#sns.set(font_scale=1)

g = sns.clustermap(corr_matrix, annot=True, fmt=".2f", cmap='coolwarm', 
                   linewidths=0.5, figsize=(16, 16), dendrogram_ratio=(.15, .15),
                   cbar=False, annot_kws={"size": 9})  # Set cbar to False

g.cax.set_visible(False)

# Add title (if needed)
g.fig.suptitle(r'\textbf{Multi-clock Spearman clustermap}', fontsize=14, y = 1.00)

# Save the figure
g.savefig("example_data/figure1a.pdf", dpi=300)

plt.show()
```

# Experiment 3: clock behaviour during a reprogramming timecourse¶

One of the most remarkable experiments done in the last few years is age-reversal through reprogramming with the Yamanaka factors OSKM. However, it is unclear how different clocks behave and if they all agree that such treatment indeed lead to the reversal of most, if not all, aging signatures. Let's check, then, how they behave.

In [48]:

```
# GSE54848
sample_dict = {
    'GSM1345997':0,
    'GSM1345998':0,
    'GSM1345999':0,
    'GSM1346000':3,
    'GSM1346001':3,
    'GSM1346002':7,
    'GSM1346003':7,
    'GSM1346004':7,
    'GSM1346005':11,
    'GSM1346006':11,
    'GSM1346007':11,
    'GSM1346008':15,
    'GSM1346009':15,
    'GSM1346010':20,
    'GSM1346011':20,
    'GSM1346012':28,
    'GSM1346013':28,
    'GSM1346014':28
}
```

In [47]:

```
dfs = []
for gsm in sample_dict.keys():
    gsm_obj = GEOparse.get_GEO(geo=gsm, destdir="example_data")
    df = gsm_obj.table
    df.index = df['ID_REF'].tolist()
    df = df.loc[:,['VALUE']]
    df.columns = [gsm]
    df = df.T
    df['day'] = sample_dict[gsm]
    dfs += [df]
df = pd.concat(dfs)
df.to_pickle('example_data/reprogramming_data_results.pkl')
```

```
03-Mar-2024 09:49:52 DEBUG utils - Directory example_data already exists. Skipping.
03-Mar-2024 09:49:52 INFO GEOparse - File already exist: using local version.
03-Mar-2024 09:49:52 INFO GEOparse - Parsing example_data/GSM1345997.txt: 
03-Mar-2024 09:49:53 DEBUG utils - Directory example_data already exists. Skipping.
03-Mar-2024 09:49:53 INFO GEOparse - File already exist: using local version.
03-Mar-2024 09:49:53 INFO GEOparse - Parsing example_data/GSM1345998.txt: 
03-Mar-2024 09:49:53 DEBUG utils - Directory example_data already exists. Skipping.
03-Mar-2024 09:49:53 INFO GEOparse - File already exist: using local version.
03-Mar-2024 09:49:53 INFO GEOparse - Parsing example_data/GSM1345999.txt: 
03-Mar-2024 09:49:54 DEBUG utils - Directory example_data already exists. Skipping.
03-Mar-2024 09:49:54 INFO GEOparse - File already exist: using local version.
03-Mar-2024 09:49:54 INFO GEOparse - Parsing example_data/GSM1346000.txt: 
03-Mar-2024 09:49:54 DEBUG utils - Directory example_data already exists. Skipping.
03-Mar-2024 09:49:54 INFO GEOparse - File already exist: using local version.
03-Mar-2024 09:49:54 INFO GEOparse - Parsing example_data/GSM1346001.txt: 
03-Mar-2024 09:49:54 DEBUG utils - Directory example_data already exists. Skipping.
03-Mar-2024 09:49:54 INFO GEOparse - File already exist: using local version.
03-Mar-2024 09:49:54 INFO GEOparse - Parsing example_data/GSM1346002.txt: 
03-Mar-2024 09:49:55 DEBUG utils - Directory example_data already exists. Skipping.
03-Mar-2024 09:49:55 INFO GEOparse - File already exist: using local version.
03-Mar-2024 09:49:55 INFO GEOparse - Parsing example_data/GSM1346003.txt: 
03-Mar-2024 09:49:55 DEBUG utils - Directory example_data already exists. Skipping.
03-Mar-2024 09:49:55 INFO GEOparse - File already exist: using local version.
03-Mar-2024 09:49:55 INFO GEOparse - Parsing example_data/GSM1346004.txt: 
03-Mar-2024 09:49:56 DEBUG utils - Directory example_data already exists. Skipping.
03-Mar-2024 09:49:56 INFO GEOparse - File already exist: using local version.
03-Mar-2024 09:49:56 INFO GEOparse - Parsing example_data/GSM1346005.txt: 
03-Mar-2024 09:49:56 DEBUG utils - Directory example_data already exists. Skipping.
03-Mar-2024 09:49:56 INFO GEOparse - File already exist: using local version.
03-Mar-2024 09:49:56 INFO GEOparse - Parsing example_data/GSM1346006.txt: 
03-Mar-2024 09:49:56 DEBUG utils - Directory example_data already exists. Skipping.
03-Mar-2024 09:49:56 INFO GEOparse - File already exist: using local version.
03-Mar-2024 09:49:56 INFO GEOparse - Parsing example_data/GSM1346007.txt: 
03-Mar-2024 09:49:57 DEBUG utils - Directory example_data already exists. Skipping.
03-Mar-2024 09:49:57 INFO GEOparse - File already exist: using local version.
03-Mar-2024 09:49:57 INFO GEOparse - Parsing example_data/GSM1346008.txt: 
03-Mar-2024 09:49:57 DEBUG utils - Directory example_data already exists. Skipping.
03-Mar-2024 09:49:57 INFO GEOparse - File already exist: using local version.
03-Mar-2024 09:49:57 INFO GEOparse - Parsing example_data/GSM1346009.txt: 
03-Mar-2024 09:49:58 DEBUG utils - Directory example_data already exists. Skipping.
03-Mar-2024 09:49:58 INFO GEOparse - File already exist: using local version.
03-Mar-2024 09:49:58 INFO GEOparse - Parsing example_data/GSM1346010.txt: 
03-Mar-2024 09:49:58 DEBUG utils - Directory example_data already exists. Skipping.
03-Mar-2024 09:49:58 INFO GEOparse - File already exist: using local version.
03-Mar-2024 09:49:58 INFO GEOparse - Parsing example_data/GSM1346011.txt: 
03-Mar-2024 09:49:59 DEBUG utils - Directory example_data already exists. Skipping.
03-Mar-2024 09:49:59 INFO GEOparse - File already exist: using local version.
03-Mar-2024 09:49:59 INFO GEOparse - Parsing example_data/GSM1346012.txt: 
03-Mar-2024 09:49:59 DEBUG utils - Directory example_data already exists. Skipping.
03-Mar-2024 09:49:59 INFO GEOparse - File already exist: using local version.
03-Mar-2024 09:49:59 INFO GEOparse - Parsing example_data/GSM1346013.txt: 
03-Mar-2024 09:49:59 DEBUG utils - Directory example_data already exists. Skipping.
03-Mar-2024 09:49:59 INFO GEOparse - File already exist: using local version.
03-Mar-2024 09:49:59 INFO GEOparse - Parsing example_data/GSM1346014.txt:
```

In [14]:

```
df = pd.read_pickle('example_data/reprogramming_data_results.pkl')
df['Homo sapiens'] = 1
adata = pya.pp.df_to_adata(df, metadata_cols=['day'], imputer_strategy='mean', verbose=False)
dnam_clocks = [
    'altumage',
    'dnamphenoage',
    'dnamfitage',
    'dnamtl',
    'dunedinpace',
    'encen100',
    'encen40',
    'grimage',
    'grimage2',
    'han',
    'hannum',
    'horvath2013',
    'hrsinchphenoage',
    'knight',
    'leecontrol',
    'leerefinedrobust',
    'leerobust',
    'lin',
    'mammalian1',
    'mammalian2',
    'mammalian3',
    'mammalianblood2',
    'mammalianblood3',
    'mammalianlifespan',
    'mammalianskin2',
    'mammalianskin3',
    'pcdnamtl',
    'pcgrimage',
    'pchannum',
    'pchorvath2013',
    'pcphenoage',
    'pcskinandblood',
    'pedbe',
    'skinandblood',
    'zhangen',
    'replitali',
    'yingcausage',
    'yingadaptage',
    'yingdamage',
]
pya.pred.predict_age(adata, dnam_clocks, verbose=False)
```

In [15]:

```
# Normalize age predictions
norm_df = adata.obs.copy()
norm_df = norm_df.astype(float)
for col in norm_df.columns:
    if col == 'day':
        continue
    norm_df[col] = (norm_df[col]-np.min(norm_df[col]))/(np.max(norm_df[col]) - np.min(norm_df[col]))
```

## Lineplot¶

In [37]:

```
matplotlib.rcParams['text.usetex'] = True
matplotlib.rcParams['font.family'] = 'serif'
matplotlib.rcParams['font.serif'] = 'Times New Roman'
matplotlib.rcParams['font.size'] = 12
```

In [38]:

```
long_df = pd.melt(norm_df, id_vars='day', var_name='Variable', value_name='Value')

# Create the line plot
plt.figure(figsize=(12, 7), dpi=300)  # Increase the figure size and specify DPI here

# Choose a color palette
palette = sns.color_palette("husl", long_df['Variable'].nunique())

# Plot the data with a chosen palette and with a line style differentiation
sns.lineplot(data=long_df, x='day', y='Value', hue='Variable', palette=palette,
             style='Variable', markers=True, dashes=False, markeredgewidth = 0.5,
             err_kws={'alpha': 0.07}, errorbar=('ci', 90))  # Decrease alpha here for lighter error bands

# Set the labels and title
plt.title(r'\textbf{OSKM reprogramming timecourse}', fontsize=14)
plt.xlabel('Day', fontsize=12)
plt.ylabel('Scaled predicted age', fontsize=12)

# Place the legend outside the plot
plt.legend(title='Clock', bbox_to_anchor=(1.01, 1), loc='upper left', fontsize=7.7)

# Adjust layout for the legend
plt.tight_layout()

# Save figure
plt.savefig('example_data/figure1d.pdf', dpi=300, bbox_inches='tight')

# Show the plot
plt.show()
```

```
/Users/lucascamillo/mambaforge/envs/kaggle_icr/lib/python3.11/site-packages/seaborn/_oldcore.py:1498: FutureWarning: is_categorical_dtype is deprecated and will be removed in a future version. Use isinstance(dtype, CategoricalDtype) instead
  if pd.api.types.is_categorical_dtype(vector):
/Users/lucascamillo/mambaforge/envs/kaggle_icr/lib/python3.11/site-packages/seaborn/_oldcore.py:1498: FutureWarning: is_categorical_dtype is deprecated and will be removed in a future version. Use isinstance(dtype, CategoricalDtype) instead
  if pd.api.types.is_categorical_dtype(vector):
/Users/lucascamillo/mambaforge/envs/kaggle_icr/lib/python3.11/site-packages/seaborn/_oldcore.py:1498: FutureWarning: is_categorical_dtype is deprecated and will be removed in a future version. Use isinstance(dtype, CategoricalDtype) instead
  if pd.api.types.is_categorical_dtype(vector):
/Users/lucascamillo/mambaforge/envs/kaggle_icr/lib/python3.11/site-packages/seaborn/_oldcore.py:1498: FutureWarning: is_categorical_dtype is deprecated and will be removed in a future version. Use isinstance(dtype, CategoricalDtype) instead
  if pd.api.types.is_categorical_dtype(vector):
/Users/lucascamillo/mambaforge/envs/kaggle_icr/lib/python3.11/site-packages/seaborn/_oldcore.py:1498: FutureWarning: is_categorical_dtype is deprecated and will be removed in a future version. Use isinstance(dtype, CategoricalDtype) instead
  if pd.api.types.is_categorical_dtype(vector):
/Users/lucascamillo/mambaforge/envs/kaggle_icr/lib/python3.11/site-packages/seaborn/_oldcore.py:1498: FutureWarning: is_categorical_dtype is deprecated and will be removed in a future version. Use isinstance(dtype, CategoricalDtype) instead
  if pd.api.types.is_categorical_dtype(vector):
/Users/lucascamillo/mambaforge/envs/kaggle_icr/lib/python3.11/site-packages/seaborn/_oldcore.py:1498: FutureWarning: is_categorical_dtype is deprecated and will be removed in a future version. Use isinstance(dtype, CategoricalDtype) instead
  if pd.api.types.is_categorical_dtype(vector):
/Users/lucascamillo/mambaforge/envs/kaggle_icr/lib/python3.11/site-packages/seaborn/_oldcore.py:1498: FutureWarning: is_categorical_dtype is deprecated and will be removed in a future version. Use isinstance(dtype, CategoricalDtype) instead
  if pd.api.types.is_categorical_dtype(vector):
/Users/lucascamillo/mambaforge/envs/kaggle_icr/lib/python3.11/site-packages/seaborn/_oldcore.py:1498: FutureWarning: is_categorical_dtype is deprecated and will be removed in a future version. Use isinstance(dtype, CategoricalDtype) instead
  if pd.api.types.is_categorical_dtype(vector):
/Users/lucascamillo/mambaforge/envs/kaggle_icr/lib/python3.11/site-packages/seaborn/_oldcore.py:1498: FutureWarning: is_categorical_dtype is deprecated and will be removed in a future version. Use isinstance(dtype, CategoricalDtype) instead
  if pd.api.types.is_categorical_dtype(vector):
/Users/lucascamillo/mambaforge/envs/kaggle_icr/lib/python3.11/site-packages/seaborn/_oldcore.py:1119: FutureWarning: use_inf_as_na option is deprecated and will be removed in a future version. Convert inf values to NaN before operating instead.
  with pd.option_context('mode.use_inf_as_na', True):
/Users/lucascamillo/mambaforge/envs/kaggle_icr/lib/python3.11/site-packages/seaborn/_oldcore.py:1119: FutureWarning: use_inf_as_na option is deprecated and will be removed in a future version. Convert inf values to NaN before operating instead.
  with pd.option_context('mode.use_inf_as_na', True):
```

## Spearman correlation between reprogramming timepoint and predicted age¶

In [101]:

```
for col in norm_df.columns:
    if col == 'day':
        continue
    print(col)
    print(spearmanr(norm_df['day'], norm_df[col]))
```

```
altumage
SignificanceResult(statistic=-0.9734713738318039, pvalue=1.1350529215821763e-11)
dnamphenoage
SignificanceResult(statistic=-0.8963440915367787, pvalue=4.819235664127294e-07)
dnamfitage
SignificanceResult(statistic=-0.8536114081030486, pvalue=6.628872080609285e-06)
dnamtl
SignificanceResult(statistic=0.5701165326402535, pvalue=0.013499179838419544)
dunedinpace
SignificanceResult(statistic=-0.35332633375693956, pvalue=0.15034907386750682)
encen100
SignificanceResult(statistic=-0.27932583317657755, pvalue=0.2616379989068026)
encen40
SignificanceResult(statistic=-0.7149907520863142, pvalue=0.0008529926883044401)
grimage
SignificanceResult(statistic=-0.6680890263663666, pvalue=0.002442769251131238)
grimage2
SignificanceResult(statistic=-0.6712158080810298, pvalue=0.0022901757954496334)
han
SignificanceResult(statistic=-0.8504846263883856, pvalue=7.769136726895047e-06)
hannum
SignificanceResult(statistic=-0.2605651428885985, pvalue=0.29634719242733226)
horvath2013
SignificanceResult(statistic=-0.9536684229722705, pvalue=9.228944453825616e-10)
hrsinchphenoage
SignificanceResult(statistic=-0.8025406400968834, pvalue=6.123600510315028e-05)
knight
SignificanceResult(statistic=-0.849442365816831, pvalue=8.184803726373807e-06)
leecontrol
SignificanceResult(statistic=-0.9797249372611305, pvalue=1.3474947909469003e-12)
leerefinedrobust
SignificanceResult(statistic=-0.9588797258300424, pvalue=3.612045013116204e-10)
leerobust
SignificanceResult(statistic=-0.9745136344033584, pvalue=8.263893996935956e-12)
lin
SignificanceResult(statistic=-0.9088512183954316, pvalue=1.794041053326807e-07)
mammalian1
SignificanceResult(statistic=-0.915104781824758, pvalue=1.0367076694792486e-07)
mammalian2
SignificanceResult(statistic=-0.8192168092417537, pvalue=3.197845705652955e-05)
mammalian3
SignificanceResult(statistic=-0.5284261097780778, pvalue=0.024169621873484124)
mammalianblood2
SignificanceResult(statistic=-0.4033548411915505, pvalue=0.09694912987796174)
mammalianblood3
SignificanceResult(statistic=-0.23138184688507546, pvalue=0.3555808639160035)
mammalianlifespan
SignificanceResult(statistic=-0.177184297164247, pvalue=0.48183272845063974)
mammalianskin2
SignificanceResult(statistic=-0.8129632458124273, pvalue=4.110327958884068e-05)
mammalianskin3
SignificanceResult(statistic=-0.9307386903980738, pvalue=2.139576956117878e-08)
pcdnamtl
SignificanceResult(statistic=0.8536114081030486, pvalue=6.628872080609285e-06)
pcgrimage
SignificanceResult(statistic=-0.9536684229722705, pvalue=9.228944453825616e-10)
pchannum
SignificanceResult(statistic=-0.8473578446737222, pvalue=9.07360822230189e-06)
pchorvath2013
SignificanceResult(statistic=-0.9640910286878145, pvalue=1.2418898796612532e-10)
pcphenoage
SignificanceResult(statistic=-0.870287577247919, pvalue=2.661401843073942e-06)
pcskinandblood
SignificanceResult(statistic=-0.9588797258300424, pvalue=3.612045013116204e-10)
pedbe
SignificanceResult(statistic=-0.9745136344033584, pvalue=8.263893996935956e-12)
skinandblood
SignificanceResult(statistic=-0.8327661966719607, pvalue=1.7942694039465786e-05)
zhangen
SignificanceResult(statistic=-0.9849362401189024, pvalue=1.271713209019279e-13)
replitali
SignificanceResult(statistic=-0.9078089578238772, pvalue=1.958268047800313e-07)
yingcausage
SignificanceResult(statistic=-0.9317809509696282, pvalue=1.90150135915936e-08)
yingadaptage
SignificanceResult(statistic=0.6065956526446573, pvalue=0.007606669731273618)
yingdamage
SignificanceResult(statistic=-0.8973863521083333, pvalue=4.4600208867136975e-07)
```

In [ ]:

```

```
